# Skin mucus of *Cyprinus carpio *inhibits cyprinid herpesvirus 3 binding to epidermal cells

**DOI:** 10.1186/1297-9716-42-92

**Published:** 2011-08-04

**Authors:** Victor Stalin Raj, Guillaume Fournier, Krzysztof Rakus, Maygane Ronsmans, Ping Ouyang, Benjamin Michel, Cédric Delforges, Bérénice Costes, Frédéric Farnir, Baptiste Leroy, Ruddy Wattiez, Charles Melard, Jan Mast, François Lieffrig, Alain Vanderplasschen

**Affiliations:** 1 Immunology-Vaccinology (B43b), Department of Infectious and Parasitic Diseases (B43b), Faculty of Veterinary Medicine, University of Liège, 4000 Liège, Belgium; 2 Biostatistics (B43), Faculty of Veterinary Medicine, University of Liège, 4000 Liège, Belgium; 3 Proteomic and protein biochemistry (Pentagone), University of Mons, 7000 Mons, Belgium; 4 CEFRA-University of Liège, 10 Chemin de la Justice, 4500 Tihange, Belgium; 5 Department Biocontrole, Research Unit Electron Microscopy, Veterinary and Agrochemical Research Centre, VAR-CODA-CERVA, Groeselenberg 99, 1180 Ukkel, Belgium; 6 CERgroupe, rue du Carmel 1, 6900 Marloie, Belgium

## Abstract

Cyprinid herpesvirus 3 (CyHV-3) is the aetiological agent of a mortal and highly contagious disease in common and koi carp. The skin is the major portal of entry of CyHV-3 in carp after immersion in water containing the virus. In the present study, we used in vivo bioluminescence imaging to investigate the effect of skin mucus removal and skin epidermis lesion on CyHV-3 entry. Physical treatments inducing removal of the mucus up to complete erosion of the epidermis were applied on a defined area of carp skin just before inoculation by immersion in infectious water. CyHV-3 entry in carp was drastically enhanced on the area of the skin where the mucus was removed with or without associated epidermal lesion. To investigate whether skin mucus inhibits CyHV-3 binding to epidermal cells, tail fins with an intact mucus layer or without mucus were inoculated ex vivo. While electron microscopy examination revealed numerous viral particles bound on the fins inoculated after mucus removal, no particle could be detected after infection of mucus-covered fins. Finally, anti-CyHV-3 neutralising activity of mucus extract was tested in vitro. Incubation of CyHV-3 with mucus extract reduced its infectivity in a dose dependent manner. The present study demonstrates that skin mucus removal and epidermal lesions enhance CyHV-3 entry in carp. It highlights the role of fish skin mucus as an innate immune protection against viral epidermal entry.

## Introduction

The koi herpesvirus (KHV), also known as cyprinid herpesvirus 3 (CyHV-3; species *Cyprinid herpesvirus 3*, genus *Cyprinivirus*, family *Alloherpesviridae*, order *Herpesvirales*), is the aetiological agent of a lethal disease in common (*Cyprinus carpio carpio*) and koi (*Cyprinus carpio koi*) carp [1-5]. Since its emergence, in the late 1990s, this highly contagious disease has caused severe economic losses in both common and koi carp culture industries worldwide [6,7].

Recently, we demonstrated using a CyHV-3 recombinant strain expressing luciferase (LUC) and in vivo bioluminescence imaging that the major portal of entry for CyHV-3 in carp after immersion in infectious water is the skin covering the fins and the body [8]. This study together with an earlier report addressing the portal of entry of the rhabdovirus *Infectious hematopoietic necrosis virus *in salmonids [9] suggest that the skin of teleost fish represents an efficient portal of entry for some viruses.

**Figure 1:**
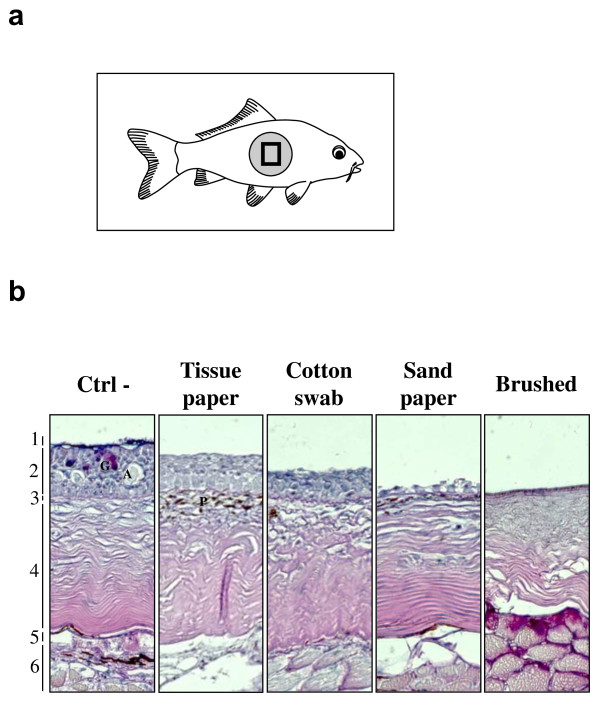
**Effect of physical treatments on fish skin**. (a) The physical treatments described in the methods were applied to the skin area indicated by a grey disc (diameter of 15 mm). Immediately after the treatment, a biopsy was made in the centre of the treated area (Black square) and processed for histological examination. (b) Histological examination of the biopsy 1: Mucus layer, G: Goblet cell, A: Alarm cell, P: chromatophore, 2: Epidermis, 3: Basement membrane, 4: Dermis, 5: Hypodermis and 6: Subcutaneous muscles.

The skin of teleost fish is made up of five structures (Figure 1b, left panel). The mucus layer or cuticle covers the epidermis [10]. The latter is a stratified squamous epithelium composed of three cell layers: (i) the superficial layer, composed of flattened squamous cells, (ii) the intermediate layer, "*stratum germinativum*", encompassing squamous and cuboidal cells and (iii) the basal layer "*stratum basale*" composed of columnar epithelial cells covering the basement membrane. Importantly, unlike its mammalian counterpart, fish epidermis is living and capable of mitotic division at all levels, even at the outermost squamous layer. The predominant cell type in the epidermis is the Malphigian cells. However, glandular cells such as goblet cells secreting mucus and club cells secreting potent alarm substances are also present. The epidermis and the dermis are separated by a relatively thick basement membrane containing pigment cells. The scales are dermis structures and consequently are covered by the epidermis.

Fish skin is a complex limiting structure providing mechanical, chemical and immune protection against injury and pathogenic microorganisms [11]. Its mucus layer confers an innate immune protection against pathogen entry. Two types of mechanisms explain the protection conferred by mucus. Firstly, the mucus forms an efficient mechanical barrier that is constantly moving downstream along the fish and off of trailing edges. Like the muco-ciliary escalator of the respiratory tract of pulmonate animals, fish mucus reduces pathogen access to epithelial cells. Secondly, the mucus contains numerous proteins such as for example immunoglobulins, enzymes and lytic agents able to neutralise microorganisms [11-15]. It is generally accepted that chemical and physical (for example, ectoparasite infestations, rude handling or injuries) stresses that affect skin mucus increase fish susceptibility to infection by pathogens [10]. However, despite the abundance of studies on fish skin immunity and skin bacterial infection, there are few in vivo evidence on the role of skin mucus as a first line of innate immune protection against bacterial infection, and none against viral infection [16-20].

In the present study, we investigated the roles of epidermal mucus as an innate immune barrier against CyHV-3 entry. Our results demonstrate that the mucus of the skin inhibits CyHV-3 binding to epidermal cells and is able to neutralise CyHV-3 infectivity.

## Materials and methods

### Cells and virus

*Cyprinus carpio *brain cells (CCB) [21] were cultured in minimum essential medium (MEM) (Invitrogen, Merelbeke, Belgium) containing 4.5 g/L glucose (D-glucose monohydrate, Merck, Darmstadt, Germany) and 10% fetal calf serum [21]. Cells were cultured at 25°C in a humid atmosphere containing 5% CO_2 _[22]. The KHV FL BAC 136 LUC TK revertant strain of CyHV-3 was described previously [8]. This recombinant strain encodes a firefly luciferase (LUC) expression cassette inserted in the intergenic region between open reading frame (ORF) 136 and ORF137. The KHV FL BAC recovered strain of CyHV-3 was described previously [22]. This recombinant strain encodes an enhanced green fluorescent protein (EGFP) expression cassette inserted at the end of ORF55.

### Fish

Koi carp *(Cyprinus carpio koi) *(Hazorea Aquatics, Kibbutz Hazorea, Israel) and common carp *(Cyprinus carpio carpio) *(CEFRA, University of Liège, Belgium), with an average weight of 16 g, were kept in 60-liter tanks at 24°C. Microbiological, parasitical and clinical examinations of the fish just before the experiments demonstrated that these fish were fully healthy.

### Physical treatments of the skin

Four physical treatments were applied on a defined area of the carp epidermis (disc shape, diameter of 15 mm): rubbing with a soft tissue paper (TORK premium, Goteborg, Sweden), rubbing with a cotton swab (Swube Applicator, Becton Dickinson Microbiology system, Maryland, USA), brushing with a rotary electric tooth brush (Philips Sensiflex HX 1513, Anderlecht, Belgium) for 2 s or rubbing with sandpaper (average particle diameter of 265 μm, Medium p60, LUX Wermelskirchen, Germany).

### Histochemistery and microscopy analysis

Fish skin explants were fixed by immersion in Carnoy solution (ethanol 6: acetic acid 1: chloroform 3, v/v/v) for 2 h at 4°C. After dehydration with ethanol, samples were embedded in paraffin [23]. Five μm thick sections were stained by a combined Alcian Blue (AB) and Periodic acid-Schiff (PAS) staining [24]. Mounted samples were observed using a Nikon Eclipse TE 2000-S microscope equipped with a DC 300F charge-coupled device (CCD) camera (Leica, Heerbrugg, Switzerland).

### Culture of tail fin explants

Fish were euthanized using benzocaine (100 mg/L of water) (Sigma-Aldrich, Saint Louis, Missouri). The ventral lobe of the tail fin was clipped with forceps before section. Fin fragments maintained in forceps were immerged in a vertical position in minimum essential medium (GIBCO, Invitrogen, Paisley, UK) containing 4.5 g/liter glucose (D-glucose monohydrate; Merck, Damstadt, Germany) and 10% fetal calf serum (FCS) (Greiner Bio One, Frickenhausen, Germany). Tail fin explants were cultured at 25°C in a humid atmosphere containing 5% CO_2_.

### CyHV-3 inoculation of carp

For viral inoculation mimicking natural infection, fish were kept for 2 h in water containing 10^3 ^plaque forming unit (PFU)/mL of the KHV FL BAC 136 LUC TK revertant strain. At the end of the incubation period, fish were returned to larger tanks. To avoid removal of skin mucus, fish were caught using a container rather than a fish net, and they were manipulated with great care wearing humidified latex gloves. The animal study was accredited by the local ethics committee of the University of Liège, Belgium (Laboratory accreditation N°1610008, protocol N°810).

### Bioluminescence imaging

Imaging of firefly (*Photinus pyralis*) LUC was performed using an "in vivo imaging system" (IVIS) (IVIS^®^spectrum, Xenogen, Caliper LifeSciences, Hopkinton, Massachusetts, USA) as described previously [8]. For in vivo analysis, fish were anesthetized with benzocaine (50 mg/L of water). Ten minutes before bioluminescence analysis, D-luciferin (150 mg/kg body weight) (Xenogen, Caliper LifeSciences, Hopkinton, Massachusetts, USA) was administrated by intraperitoneal injection. Each fish was analyzed lying on its left and right side. For analysis of tail fin explants cultured ex vivo, culture medium was replaced by fresh medium containing D-luciferin (150 μg/mL) ten minutes before bioluminescence analysis. All the images presented in this study were acquired using a field view of 15 cm, a 1 min exposure time, a binning factor of 4 and a f/stop of 1. Relative intensities of transmitted light from bioluminescence were represented as a pseudocolor image ranging from violet (least intense) to red (most intense). Corresponding grey-scale photographs and color luciferase images were superimposed using the LivingImage analysis software (Xenogen, Caliper LifeSciences, Hopkinton, Massachusetts, USA).

### Transmission electron microscopy

Samples were fixed in 0.1% glutaraldehyde (Sigma-Aldrich, Saint Louis, Missouri, USA). Epon blocks and sections were prepared as described elsewhere [25]. Sections were analyzed using a Tecnai Spirit transmission electron microscope (FEI, Eindhoven, The Netherlands), and electron micrographs were taken using a bottom-mounted 4-by-4 K Eagle camera (FEI).

### Collection of carp epidermal mucus and production of clarified mucus extract

Epidermal mucus was collected from common carp (average weight of 5 kg) kept at 22°C (CEFRA, University of Liège, Belgium). Immediately after euthanasia, epidermal mucus was collected by gentle scraping of fish flanks using a soft rubber spatula. Mucus samples were pooled and stored on ice. Clarified mucus extract (CME) was then prepared as follows. Mucus was first clarified by centrifugation (2000 *g *for 10 min at 4°C). Clarified mucus was diluted five times in MEM on ice. To enhance mucus solubilisation, β2-mercaptoethanol (Sigma-Aldrich) was added at the final concentration of 5 mM. The sample was then processed five times through a 7 mL Dounce homogenizer (tight pestle, VWR, Chicago, USA). After an incubation of 30 min on ice, the sample was ultracentrifuged at 100 000 *g *for 30 min at 4°C. The supernatant was collected and sterilized by filtration through a 0.45 μm filter (0.45 μm filter PES, VWR). Finally, the sample was concentrated five times by centrifugation through an Amicon Ultra 3K column (Millipore). The resulting product, hereafter called CME, was stored at -80°C until use. The CME used in the present study had an estimated protein concentration of 0.95 mg/mL as determined with the non-interfering protein assay (GBiosciences, St Louis, USA).

### CyHV-3 neutralisation assay by CME

The KHV FL BAC recovered strain of CyHV-3 was diluted in MEM to reach a concentration of 5.10^4 ^plaque forming unit (pfu)/mL. The effect of CME on CyHV-3 infectivity was tested under two conditions hereafter called pre-incubation and post-incubation addition of CME. For pre-incubation addition of CME, the virus suspension was mixed with adequate volumes of CME and MEM supplemented with 5 mM β2-mercaptoethanol to reach CME final concentrations (vol/vol) of 1/2, 1/4, 1/8, 1/16 and 1/32. Samples were then incubated at 25°C for 2 h. For post-incubation addition of CME, the samples were processed as described above with the exception that the CME volumes were added after the 2 h incubation period. A negative control (NC) sample consisted of incubating the viral suspension with an equal volume of MEM supplemented with 5 mM β2-mercaptoethanol before the 2 h incubation period. All samples were then diluted 200 times in MEM and CyHV-3 infectivity was titrated on CCB monolayers grown in 24 well plates (BD, Erembodegen, Belgium) as described elsewhere [8]. Viral plaques were counted 3 days post-infection (dpi) using an epifluorescent microscope (Eclipse TE2000-S, Nikon). Statistical analyses of the results were performed by post hoc tests on least squares means for pair wise group comparisons. These analyses were done using SAS version 9.1.

## Results

### Physical treatments applied to carp epidermis

The goal of the present study was to investigate the effects of epidermal mucus removal and progressive epidermal abrasion on CyHV-3 entry in carp. To reach that goal, four different physical treatments were applied on the defined area of carp skin as depicted in Figure 1a. To avoid an effect on untreated areas, fish were handled with care by the head and the superior lobe of the tail fin. Immediately after treatment, the centre of the treated area was submitted to histological examination (Figure 1b). Gentle rubbing of the epidermis with a soft tissue paper induced removal of the mucus without apparent damage of the epithelial cells. In contrast, the use of a cotton swab induced removal of the mucus and the upper most layers of epidermal cells. After rubbing with sand paper, only a few epidermal columnar cells were left on the basement membrane; while all cells were removed after brushing with an electric tooth brush.

### Effect of carp epidermis lesion on CyHV-3 entry in carp

**Figure 2:**
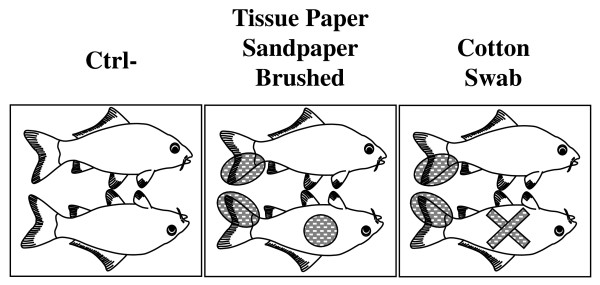
**Localised skin physical treatments applied to the fish skin just before inoculation**. Schematic diagram representing the area (in grey) of the fish skin to wish the indicated physical treatments were applied just before viral inoculation of the fish analysed in figure 3. Each panel represents the same fish lying on its left and right side.

**Figure 3:**
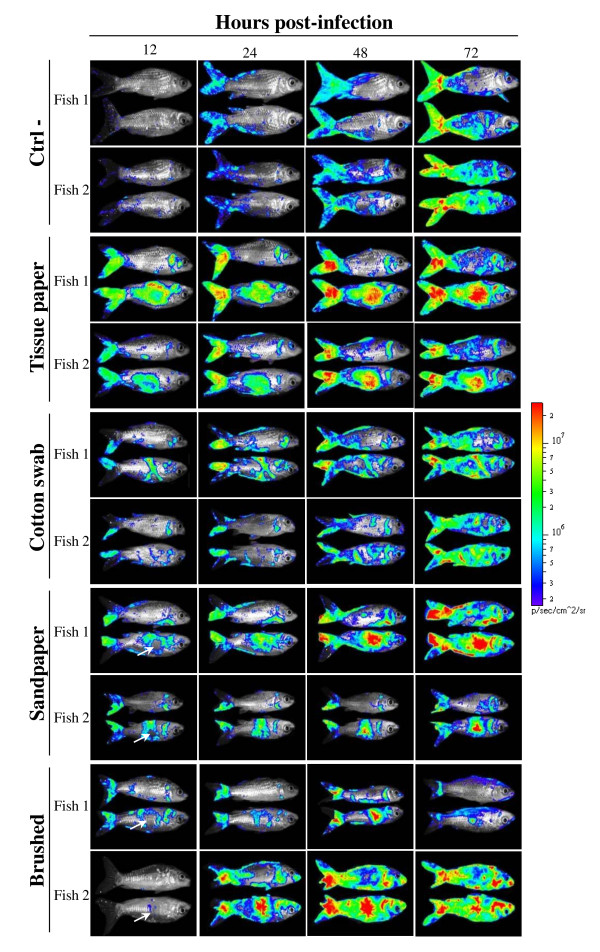
**Effect of skin physical treatments on CyHV-3 entry in carp analyzed by bioluminescence imaging**. Each physical treatment depicted in Figure 2 was applied to a group of 7 fish. Immediately after skin treatment, fish were inoculated by immersion in water containing the FL BAC 136 LUC TK revertant strain (10^3 ^PFU/mL of water for 2 h) to mimic natural infection. The fish were analyzed by bioluminescence imaging at the indicated time post-inoculation. Each fish was analyzed lying on its right and its left side. Two representative fish are shown per group. White arrows indicate the centre of epidermis lesions which was associated with no bioluminescent signal at 12 h post-inoculation but an intense signal later during infection. The images collected over the course of the experiment are presented with standardized minimum and maximum threshold values for photon flux.

The results presented above demonstrated that the different physical treatments applied locally on carp skin resulted in progressive damaging of the epidermis. These treatments were used to investigate the effect of epidermal mucus removal and progressive epidermal abrasion on CyHV-3 entry in carp. Carp skin was treated on a defined area (Figure 2) just before inoculation with the CyHV-3 KHV FL BAC 136 LUC TK revertant strain expressing LUC as a reporter gene. Sites of CyHV-3 entry in carp were revealed by IVIS examination of carp at different times post-inoculation (Figure 3).

Mucus removal and superficial abrasion of carp epidermis induced by rubbing with soft tissue paper and cotton swab enhanced CyHV-3 entry in carp. As early as 12 h post-inoculation, a strong LUC signal correlated with the area of the skin treated (Figure 3). Similarly to fish of the control group, the treated fish exhibited small foci of LUC emission distributed randomly revealing entry of the virus through unaffected skin as described earlier. According to post-inoculation time, the spread of the infection on the skin was observed as well as an increase of light emission for a determined site of infection.

**Figure 4:**
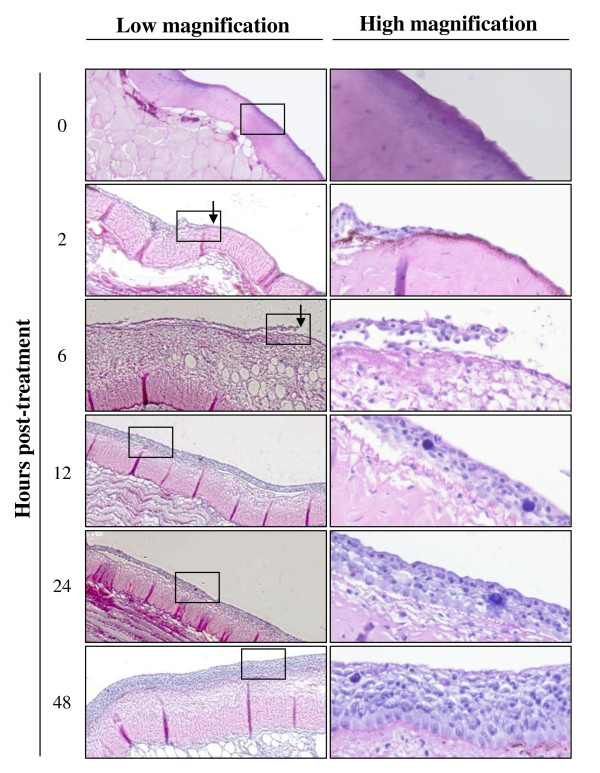
**Kinetic of epidermis healing in carp**. At time 0, the mucus and the epidermis of the skin were removed by brushing with a rotary electric tooth brush on a 15 mm circular area located on the side of the fish body. At the indicated time post-lesion, a biopsy was performed at the centre of the treated area, and processed for histological examination. Right panels represent higher magnification of the area marked in the left panels. The arrows indicate migration of epidermal cells towards the wound centre.

Deep abrasion of skin epidermis induced on the flank of fish correlated at 12 h post-inoculation with no LUC signal at the centre of the lesion while the edge of the lesion expressed LUC activity (Figure 3, Sandpaper, Brushed). The absence of LUC activity at the centre of the lesion can be explained by the removal of sensitive cells induced by the treatment; while the presence of a signal at the edge most probably resulted from mucus removal and superficial epidermis abrasion induced at the periphery of the treated area. Interestingly, starting at 24 h post-inoculation a LUC signal appeared at the centre of the treated area while it was negative 12 h earlier. This result can only be explained by an extremely fast regeneration of the epidermis throughout the centre of the lesion providing sensitive cells for viral infection. To address this hypothesis, the kinetics of epidermis healing was investigated after epidermis excoriation on a 15 mm diameter disc (Figure 4). Histological examination performed immediately after lesion induction confirmed the excoriation of the epidermis leaving the basement membrane exposed to water (Figure 4, time 0). Surprisingly, as early as 2 h post-lesion, cell migration was observed from the edge of the lesion toward its centre. The cell migration front consisted of a cell monolayer, while the number of cell layer increased progressively moving away from the centre of the lesion. At 6 h post-lesion, the migration front was nearly closing the wound. At 12 h post-lesion, the epidermis was entirely covering the basement membrane and was uniformly composed of 5-7 layers of epidermal cells with no obvious polarization of the epithelium. At 24 h post-lesion; the polarization of the epidermis was back to normal with the exception of the number of cell layers which was still inferior to normal. At 48 h post-lesion, the epidermis of the treated area could not be differentiated from the control undamaged epidermis.

Independently of the treatment applied locally to the skin, treated fish had more LUC emission foci located on the head than fish from the control group (Figure 3). This observation is likely to be the consequence of mucus removal on the head when handling the fish. Light emission was not detected from mock-infected carp used as negative controls (data not shown).

### Removal of epidermal mucus enhances CyHV-3 binding to epidermal cells

**Figure 5:**
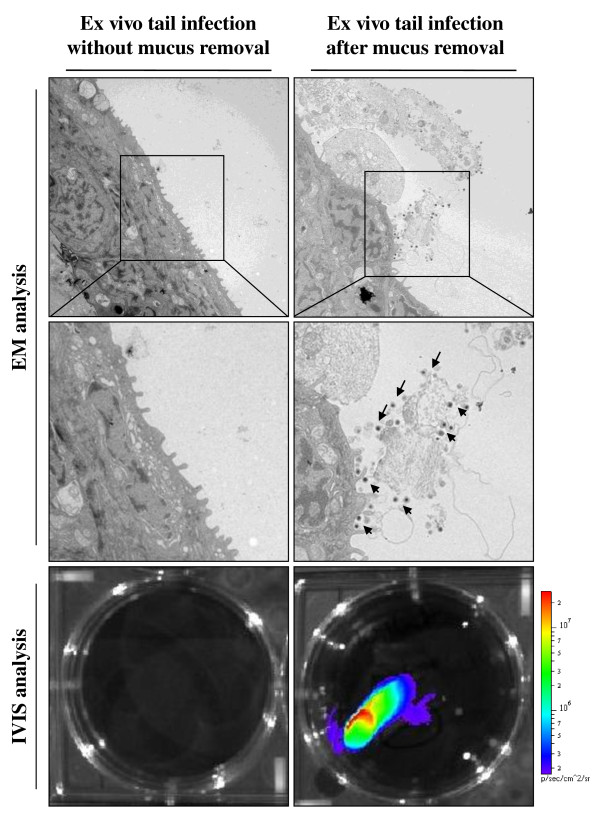
**Effect of skin mucus removal on CyHV-3 binding to carp epidermal cells**. Tail fin ventral lobes of carp were mock-treated or treated by rubbing with a soft tissue paper to removal epidermal mucus (see methods). Immediately after skin treatments, tail fin explants were harvested and inoculated ex vivo with the FL BAC 136 LUC TK revertant strain (10^6 ^PFU/mL of culture medium for 2 h). At the end of the 2 h inoculation period, a fragment of the fin was collected and processed for electron microscopy examination (EM analysis). The arrows indicate CyHV-3 particles bound to cells or cell debris. Twenty-four hours post-inoculation, duplicate tail explant cultures were analyzed by bioluminescence imaging (lower panels).

The results presented above demonstrated that removal of epidermal mucus enhances the entry of CyHV-3 in carp. This observation led to the hypothesis that epidermal mucus could act as an innate immune protection reducing CyHV-3 binding to epidermal cells. To test this hypothesis, tail fin explants with or without mucus were inoculated ex vivo with CyHV-3 (Figure 5). After an incubation of 2 h, viral binding to epidermal cells was investigated by electron microscopy examination. While no viral particles could be detected on fin explants with an intact mucus layer, numerous viral particles were observed on the surface of the fin infected after removal of mucus. Virus particles were found attached to structurally normal cells but also to lysed cells and cell debris still attached to the epidermis by desmosomes. As damaged cells were not observed in the control untreated sample (without removal of the mucus), they were thought to be the consequence of the mucus removal procedure. IVIS analysis of duplicate fin explants 24 h after inoculation confirmed that CyHV-3 infection of carp skin was drastically enhanced by mucus removal just before inoculation (Figure 5, bottom panels).

### Epidermal mucus neutralises CyHV-3 infectivity

**Figure 6:**
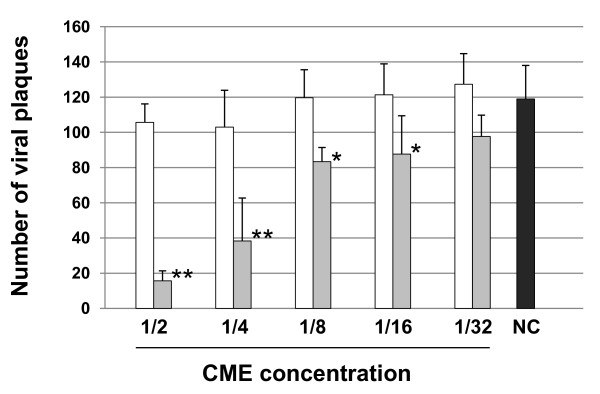
**Effect of CME on CyHV-3 infectivity**. CME was prepared from common carp epidermal mucus and tested for its ability to neutralise CyHV-3 as described in the materials and methods. Grey and white bars represent the results obtained when adding CME to the samples before and after the 2 h incubation period, respectively. NC represents the negative control sample in which no CME was added. The data presented are the means ± SE of triplicate measurements. The means that are significantly different from the mean of the NC group are marked (* ≤ 0.05, ** ≤ 0.0001).

In the last section of this study, we investigated whether epidermal mucus can neutralise CyHV-3 infectivity (Figure 6). CME was prepared from epidermal mucus and tested for its ability to neutralise CyHV-3 as described in the materials and methods. Incubation of CyHV-3 with CME at the concentration (vol/vol) of 1/2 down to 1/16 led to a statistically significant reduction of the number of viral plaques compared to the NC sample (Figure 6, pre-incubation addition of CME). In contrast, none of the concentrations tested led to a significant neutralisation effect when CME was added to the sample after the incubation period (Figure 6, post-incubation addition of CME). The latter results demonstrate that diluted CME present in both types of samples (pre- and post-addition of CME) during the final titration step did not influence CyHV-3 infectivity significantly.

## Discussion

Mucus covering fish surfaces exposed to water acts as an innate and adaptive first line of defence against pathogen entry [13]. Only very few studies addressed in vivo the role of epidermal mucus as an innate immune protection against bacterial infections [16-20]; while no study has demonstrated so far its role in preventing viral entry in fish. Here, we took advantage of the "CyHV-3 - carp" model of infection to investigate by using bioluminescence imaging the effect of mucus removal and progressive epidermal lesions on CyHV-3 entry in carp. The data of the present study demonstrated that epidermal mucus inhibits CyHV-3 binding to epidermal cells at least partially by neutralisation of viral infectivity, and that epidermal lesions enhance CyHV-3 entry in carp.

Carp epidermal mucus inhibits CyHV-3 binding on epidermal cells (Figure 5). As mentioned in the introduction, epidermal mucus confers an innate immune protection against pathogen entry. This protection relies on mechanical reduction of pathogen access to epidermal cells and eventually on pathogen neutralisation by active molecules [13]. The results presented in Figure 6 demonstrated that epidermal mucus neutralises CyHV-3 in a dose dependent manner. Fish epidermal mucus contains a growing list of molecules that could contribute to virus neutralisation, such as for example complement factors, C-reactive protein, immunoglobulines, lectins and defensins [11-15,26,27]. Future studies are required to determine the mechanisms by which epidermal mucus neutralises CyHV-3.

Despite the ability of skin mucus to inhibit CyHV-3 binding to epidermal cells, immersion of carp in infectious water led to viral entry in carp through the skin (Figure 3, Ctrl-). Two hypotheses that are not mutually exclusive can conciliate these observations. Firstly, it is likely that the inhibition of virus binding to epidermal cells by mucus is partial rather than total. Secondly, the sites of primary skin infection could represent areas of the fish body that are uncovered by mucus [13] or covered by a thinner layer compared to the rest of the body. The heterogeneity of the thickness of the mucus layer over the surface of the fish could represent physiological differences or be the consequence of mucus removal caused by physical contact. Consistent with the latter hypothesis, we observed that the sites of primary infection are mainly located at the periphery of the fins (Figure 3).

Mucus removal and epidermal lesions enhance CyHV-3 entry in carp (Figure 3). The results of the present study suggest that skin lesions caused for example by ectoparasite infestations, rough handling or inappropriate environment (as for example a tank with abrasive walls) should enhance the entry of CyHV-3 through the skin and consequently the spread of the disease. At the early stage of the disease, CyHV-3 replicates at the portal of entry [8]. This early replication in the skin probably explains why infected fish rubbed themselves against each other or against objects. This behaviour could represent an efficient "skin-to-skin" mode of transmission of CyHV-3 in the carp population by inducing physical contact between the skin of infected and naive carp with simultaneous removal of mucus. This hypothesis could at least partly explain the higher transmission dynamics of CyHV-3 in wildlife between adult carps during the host breeding season [28].

In conclusion, the present study demonstrates the role of fish epidermal mucus as an innate immune protection against a viral infection. This study further supports the role of epidermal mucus as an important component of fish innate immunity. It also provides a model to study the effect of immunostimulants on this component of fish innate immunity.

## Competing interests

The authors declare that they have no competing interests.

## Authors' contributions

VSR, GF and BC participated in the design of the study. VSR, GF, MR, PO, KR and BM performed the experiments and drafted the figures. BC coordinated some of the experiments. CM and FL controlled the sanitary statue of the carp and took care of zootechnique aspects. CD elaborated ex vivo culture of carp fins. JM performed electron microscopy analyses. FF performed statistical analyses. BL and RW produced and characterized CME. AV conceived the study and drafted the manuscript. All authors read and approved the final manuscript.
